# Nanocarbon-Driven Recovery of Mechano-Kinetic Properties of Injured Rat Gastrocnemius Muscle

**DOI:** 10.3390/ijms26125511

**Published:** 2025-06-09

**Authors:** Dmytro Nozdrenko, Yuriy Prylutskyy, Oleksii Sulyma, Yevhenii Kozik, Igor Vareniuk, Uwe Ritter, Tetiana Abramovych, Inna Sokolowska, Andriy Maznychenko

**Affiliations:** 1 ESC “Institute of Biology and Medicine”, Taras Shevchenko National University of Kyiv, Volodymyrska Str. 64, 01601 Kyiv, Ukraine; dmytro.nozdrenko@knu.ua (D.N.); prylut@ukr.net (Y.P.); vareniuk_igor@yahoo.com (I.V.); 2 State Institution “Institute of Traumatology and Orthopedics”, National Academy of Medical Science of Ukraine, Bulvarno-Kudriavska Str. 27, 01054 Kyiv, Ukraine; sulymaoleksii@gmail.com (O.S.); yevheniikozik@gmail.com (Y.K.); 3 Institute of Chemistry and Biotechnology, Technical University of Ilmenau, Weimarer Str. 25, 98693 Ilmenau, Germany; uwe.ritter@tu-ilmenau.de; 4 Department of Physical Education, Gdansk University of Physical Education and Sport, Kazimierza Gorskiego Str. 1, 80-336 Gdansk, Poland; tetiana.abramovych@awf.gda.pl (T.A.); inna.sokolowska@awf.gda.pl (I.S.); 5 Department of Movement Physiology, Bogomoletz Institute of Physiology NAS of Ukraine, Bogomoletz Str. 4, 01024 Kyiv, Ukraine

**Keywords:** C_60_ fullerene aqueous solutions, muscle mechano-kinetic properties, biochemical markers, gastrocnemius muscle, rat

## Abstract

Traumatic muscle injuries often lead to prolonged functional impairments due to oxidative stress, metabolic disturbances, and structural damage. This study investigates the therapeutic potential of chronic administration of C_60_ fullerene aqueous (C_60_FAS) solutions in enhancing muscle recovery post-injury. Rats with experimentally induced gastrocnemius muscle trauma received C_60_FAS orally at a daily dose of 1 mg/kg body weight. Functional assessments included measurements of maximal force generation and time to peak contraction. Biochemical analyses evaluated lactate, superoxide dismutase (SOD), and catalase (CAT) levels, while histological examinations assessed muscle fiber integrity and collagen deposition. Results demonstrated significant improvements in muscle function, with a 35–40% increase in maximal force and a 27–38% acceleration in contraction time. Biochemical analysis revealed a 25% decrease in lactate concentration, potentially indicating improved metabolic function. This change is in line with normalized SOD and CAT activities, suggesting enhanced redox balance following treatment. Histological analyses revealed preserved myofibrillar architecture and reduced fibrosis in treated muscles. These findings suggest that C_60_ fullerene facilitates muscle recovery through antioxidant protection, metabolic support, and structural preservation, highlighting its potential as a therapeutic agent for muscle injuries.

## 1. Introduction

Muscle injuries represent a pervasive clinical challenge, affecting athletes, military personnel, and aging populations alike. Despite their high incidence, treatment strategies remain largely palliative, hampered by the complex interplay of inflammation, oxidative stress, and fibrosis that hinders effective regeneration [1]. The resultant chronic inflammation–fibrosis axis leads to contractures and functional deficits, with current therapies failing to restore preinjury mechano-kinetic properties [2,3]. Animal models of partial-thickness muscle incision (e.g., gastrocnemius) recapitulate this pathology, revealing persistent deficits in tetanic force and fatigue resistance due to collagenous scarring [4,5]. Conventional treatments, such as non-steroidal anti-inflammatory drugs and corticosteroids, primarily offer symptomatic relief and may not adequately address the underlying pathophysiological mechanisms, i.e., excessive reactive oxygen species (ROS) and dysregulated immune responses, sometimes leading to adverse side effects [6]. This therapeutic gap has spurred interest in nanobiotechnology, where engineered nanoparticles (NPs) offer precision modulation of pathological processes at the molecular level [7]. Among these, carbon-based nanomaterials, particularly C_60_ fullerenes, stand out for their unique physicochemical properties, including exceptional electron-accepting capacity, and the ability to ROS scavenge C_60_’s ability to scavenge multiple free radicals—up to six electrons per molecule—exceeds the stoichiometric limits of conventional antioxidants (e.g., vitamin E or N-acetylcysteine), enabling sustained ROS neutralization even at low doses [8,9]. This attribute is particularly beneficial in the context of muscle injuries, where oxidative stress plays a pivotal role in secondary tissue damage. Studies have demonstrated that C_60_ fullerenes can mitigate oxidative damage, reduce inflammation, and promote tissue regeneration [5,10]. Furthermore, C_60_ fullerenes have been shown to preserve mitochondrial function, inhibit profibrotic signaling pathways such as TGF-β/Smad, modulate immune responses by attenuating NLRP3 inflammasome activation [11], and even increase the force productivity of skeletal muscles after glyphosate poisoning [12]. These multifaceted actions position C_60_ fullerenes as promising candidates for enhancing muscle repair and functional recovery following injury. Therefore, gaining an in-depth insight into the molecular and cellular processes that drive skeletal muscle adaptation in response to various stressors can enhance existing knowledge in muscle physiology. This understanding may also pave the way for novel therapeutic strategies aimed at promoting tissue regeneration and recovery [13].

In our previous study [5,10], we utilized a traumatic muscle injury model to examine the effects of the C_60_ fullerene aqueous solution (C_60_FAS), administered chronically over a two-week period, on key time-dependent mechano-kinetic parameters of muscle function. These parameters included the duration of sustained maximal muscle contraction, the relaxation time required for the muscle to return to its baseline state after stimulation, and the difference between initial and peak (maximal vs. minimal) muscle contraction forces during specific stimulation patterns. Animals treated with the fullerene solution exhibited significant improvements across all contraction parameters. We hypothesized that C_60_ fullerene, acting as an antioxidant agent, would enhance post-injury muscle recovery, particularly in contraction dynamics critical for precise movement control, such as the time to reach maximal and minimal contractile force. Furthermore, we propose that extended treatment (up to 45 days) could optimize the restoration of muscle function to near pre-injury levels.

Therefore, the aim of this study was to investigate the therapeutic potential of aqueous C_60_FAS in facilitating the recovery of posttraumatic mechano-kinetic properties, specifically focusing on the maximum force developed and the time to reach maximum contraction force by the muscle during certain patterns of electrical stimulation of the gastrocnemius muscle in a rat model of muscle injury. The experimental design involved inducing controlled transverse injuries to the muscle, followed by treatment with C_60_ fullerenes. Recovery was assessed through tensometric measurements, biochemical assays evaluating oxidative stress markers (superoxide dismutase (SOD), catalase (CAT), and lactate), and histological analyses over intervals of 15, 30, and 45 days.

## 2. Results

### 2.1. Control Muscle Performance

**Figure 1:**
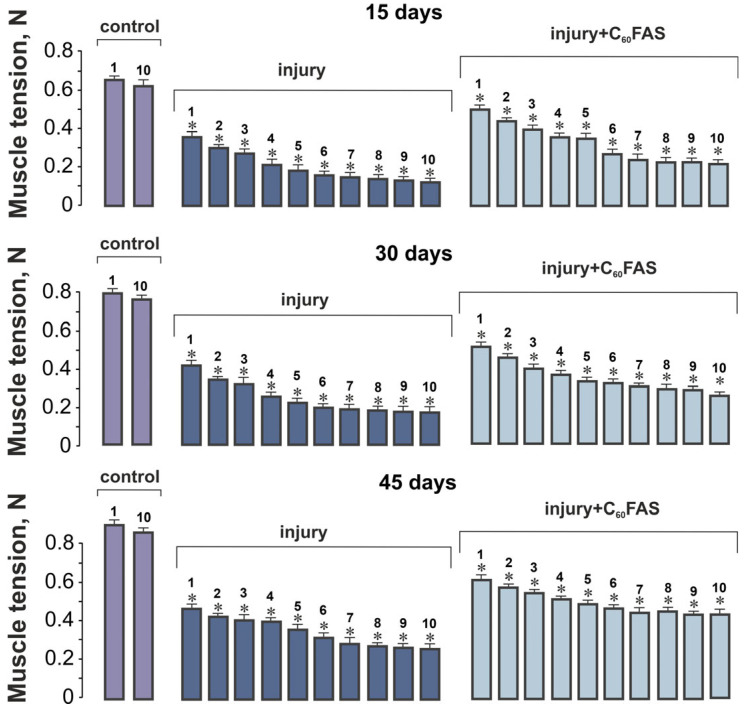
Muscle tension of rat gastrocnemius muscle following trauma and C_60_FAS treatment. Graph shows mean ± S.E.M. values for maximal contraction force generation (N, Newton) across 10 consecutive tetanic contractions (contractions 1–10 shown). Control data display values for the 1st and 10th contractions only. Experimental groups include injury (untreated trauma) and injury + C_60_FAS (trauma animals receiving daily oral administration of 1 mg/mL C_60_FAS). Time points represent 15, 30, and 45 days post-injury. Statistical significance (* *p* < 0.05 vs. control).

Control measurements of maximal muscle force in control animals (Groups 1–3: intact; Groups 4–6: sham-treated with C_60_FAS) demonstrated stable values throughout the experimental period (15 days: 6.4 ± 0.05 N; 30 days: 7.8 ± 0.06 N; 45 days: 8.9 ± 0.07 N), with no statistically significant differences between these groups at any timepoint (*p* > 0.05). This consistency allowed us to combine these groups as a unified control for subsequent comparative analyses (Figure 1).

### 2.2. Muscle Contraction Force Generation

The impact of muscle injury became evident when examining force generation patterns. At 15 days post-injury, injured animals exhibited severely compromised contractile performance, generating only 58 ± 3% of control force values during the first stimulation series, with further decline to 38 ± 2% by the tenth series. Remarkably, daily administration of aqueous C_60_FAS significantly ameliorated these deficits, restoring force generation to 91 ± 4% and 62 ± 3% of control levels for the respective stimulation series (*p* < 0.05 for both comparisons). This therapeutic effect showed progressive enhancement with longer treatment duration. By day 30, injured, untreated muscles reached only 43 ± 2% of control force capacity, while C_60_FAS-treated animals achieved 83 ± 4% recovery. Similarly, at 45 days post-injury, the injury-only group remained at 37 ± 2% of control values, whereas C_60_FAS treatment enabled 52 ± 2% functional recovery (Figure 1).

### 2.3. Temporal Dynamics of Muscle Contraction

**Figure 2:**
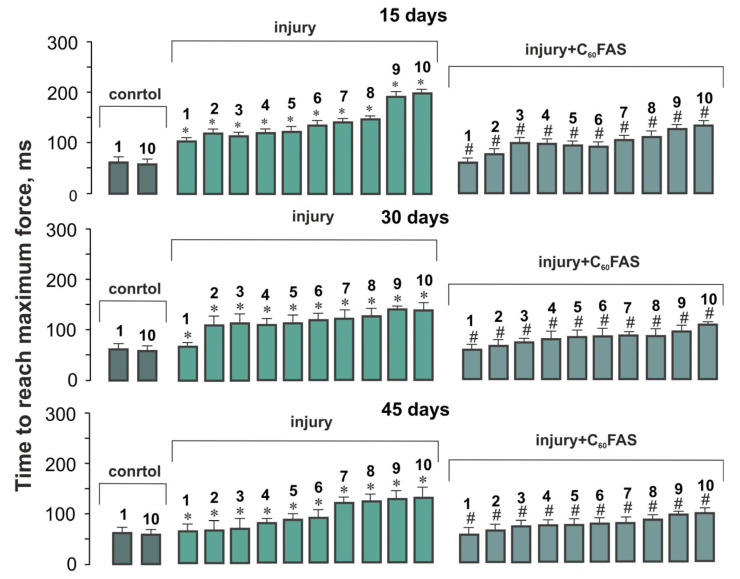
Temporal dynamics of contraction kinetics in rat gastrocnemius muscle following trauma and C_60_FAS treatment. Graph shows mean ± S.E.M. values for time to peak force (ms) across 10 consecutive tetanic contractions (contractions 1–10 shown). Control data display values for the 1st and 10th contractions only. Experimental groups include injury (untreated trauma) and injury + C_60_FAS (trauma animals receiving daily oral administration of 1 mg/mL C_60_FAS). Time points represent 15, 30, and 45 days post-injury. Statistical significance (* *p* < 0.05 vs. control; # *p* < 0.05 vs. injury group).

The temporal characteristics of muscle contraction revealed another dimension of C_60_’s therapeutic action. In control animals, the time required to reach maximal contraction force remained constant at approximately 60 ms throughout all stimulation series. Muscle injury substantially disrupted this parameter, particularly at 15 days post-trauma, where the activation time increased to 107 ms for the first contraction and reached 199 ms by the tenth series, representing 1.8- and 3.4-fold prolongations, respectively. C_60_FAS treatment effectively mitigated these delays, reducing the values to 62 ± 5 ms (first contraction) and 110 ± 10 ms (tenth contraction), corresponding to 31–38% improvements compared to untreated injured animals (Figure 2). These findings suggest that C_60_FAS not only enhances force generation capacity but also improves the speed of neuromuscular activation following injury (especially noticeable on day 45 of the experiment).

### 2.4. Systemic Biochemical Markers of Muscle Recovery

To elucidate the mechanisms underlying the observed functional improvements, we conducted comprehensive analyses of plasma biomarkers associated with muscle injury and oxidative stress.

**Figure 3:**
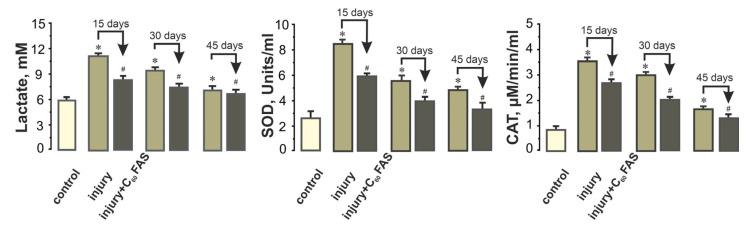
Concentrations of lactate and indicators of pro- and antioxidant balance (SOD, CAT) in the plasma of injured rats and rats receiving C_60_FAS after injury. Control, injury, and injury + C_60_FAS—indices of control group, groups of injured rats, and rats who daily consumed orally C_60_FAS at a dose of 1 mg/mL after the initiation of injury; 15, 30, and 45 days—days after the initiation of open muscle injury, respectively; * *p* < 0.05 relative to control group; # *p* < 0.05 relative to injury group.

Lactate dynamics similarly demonstrated treatment benefits. While control animals maintained lactate at 5.9 ± 0.50. mM, injury caused an immediate increase to 11.3 ± 0.7 mM at 15 days, with subsequent decline to 7.4 ± 0.8 mM by 45 days. C_60_FAS-treated animals showed a significant decrease in lactate clearance, achieving values of 8.5 ± 0.5 mM (15 days), 7.3 ± 0.4 mM (30 days), and 6.9 ± 0.5 mM (45 days)—reflecting approximately 25% greater oxidation capacity compared to their untreated counterparts (Figure 3).

Oxidative stress markers provided further mechanistic insights:

SOD activity surged to 8.1 ± 0.2 U/mL (15 days post-injury) vs. 2.6 ± 0.4 U/mL in controls, indicating substantial oxidative challenge. C_60_FAS treatment reduced this elevation by 35–40% across all timepoints (15 days: 5.9 ± 0.2 U/mL; *p* < 0.05).

CAT activity followed a similar pattern, increasing from 0.9 ± 0.1 µM/min (control) to 3.4 ± 0.1 µM/min post-injury (15 days), with C_60_FAS treatment producing 25–35% reductions (15 days: 2.4 ± 0.2 µM/min; Figure 3) and a decrease in the following days.

These biochemical findings collectively demonstrate that C_60_ fullerene exerts significant antioxidant effects, attenuating injury-induced oxidative stress and facilitating metabolic recovery.

### 2.5. Histopathological Correlates of Functional Recovery

**Figure 4:**
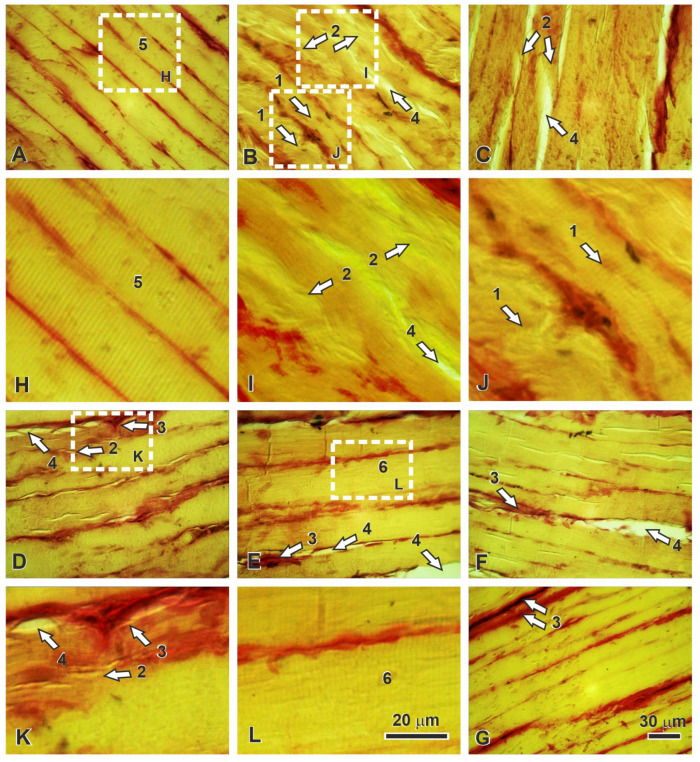
Representative micrographs of gastrocnemius muscle: (**A**)—control; (**B**)—15 days after trauma; (**C**)—15 days after trauma and C_60_FAS-treatments; (**D**)—30 days after trauma; (**E**)—30 days after trauma and C_60_FAS-treatments; (**F**)—45 days after trauma; (**G**)—45 days after trauma and C_60_FAS-treatments. The rectangular regions enclosed by dashed lines in (**A**,**B**,**D**,**E**) are shown in (**H–L**) at higher magnification. 1—tortuous muscle fibers; 2—fibrilization of muscle fibers; 3—expansion of collagen fibers; 4—ground substance of connective tissue; 5—normal cross-striation; 6—poorly visible cross-striation. Hematoxylin and picrofuchsin staining by van Gieson. Scale bars for sections (**A**–**G**)—30 μm, for (**H**–**L**)—20 μm.

Histological examination provided structural confirmation of the functional and biochemical improvements observed with C_60_FAS treatment. Control muscles displayed characteristic polygonal fibers with well-defined transverse striation and minimal endomysial connective tissue (Figure 4A).

The temporal progression of injury and recovery revealed distinct treatment effects:

Fifteen days post-injury: Untreated muscles showed extensive fiber destruction, loss of striation, and marked endomysial edema (Figure 4B). C_60_FAS-treated specimens exhibited significantly better preservation of fiber architecture, with maintained striation patterns and reduced connective tissue infiltration (Figure 4C).

Thirty days: While untreated muscles displayed persistent fiber tortuosity and developing fibrosis (Figure 4D), C_60_FAS-treated samples showed more organized regeneration with fewer pathological fibers (Figure 4E).

Forty-five days: The regeneration process in untreated animals remained incomplete, with numerous thin fibers and substantial collagen deposition (Figure 4F). In contrast, C_60_FAS-treated muscles approached near-normal histology, with only minimal residual fibrosis (Figure 4G).

This histological progression correlates precisely with the functional recovery timeline, demonstrating that C_60_FAS not only accelerates structural regeneration but also enhances the quality of muscle repair.

## 3. Discussion

This study demonstrates that chronic administration of aqueous C_60_FAS solutions significantly enhances functional recovery following traumatic muscle injury through multimodal mechanisms involving antioxidant protection, metabolic modulation, and structural preservation.

The observed improvements in muscle function and redox balance following C_60_FAS administration may be attributed to several interrelated mechanisms such as direct ROS scavenging, mitochondrial protection, and anti-inflammatory effects. The molecular mechanism underlying the antioxidant reactions of C_60_ compounds is that electron-deficient regions of C_60_ cooperate with the attached malonyl groups to electrostatically guide and stabilize superoxide, thereby promoting its decomposition [14,15]. Additionally, C_60_ nanoparticles have been shown to be selectively internalized by cerebral endothelial cells damaged by oxidative stress, significantly inhibiting apoptosis induced by ROS. This protective effect is associated with the modulation of the c-Jun N-terminal kinase signaling pathway [16]. Furthermore, some studies have demonstrated that fullerenes can significantly alleviate ROS-dependent neuronal injury induced by NMDA or potassium deficiency in vitro [17,18]. The neuroprotective effects of fullerenes have also been validated in models of cortical infarction induced by ischemia–reperfusion [15,19]. These findings suggest that the structure of C_60_ enables efficient electron acceptance, allowing it to neutralize multiple ROS, which aligns with our observed reductions in oxidative stress markers, such as SOD and CAT activity. Moreover, the strong electron affinity of fullerene materials supports their role in preventing excess electrons in the electron transport chain by functioning as a capacitor-like electron sink. Their electrophilic nature stimulates the Nrf2/ARE pathways, leading to an antioxidant response and mitochondrial biogenesis, thereby helping to maintain mitochondrial homeostasis [20]. This mechanism may explain the improved lactate metabolism observed in our study, as preserved mitochondrial function shifts energy production toward oxidative phosphorylation. Additionally, C_60_ has been reported to indirectly inhibit NF-κB activation, potentially reducing the production of pro-inflammatory cytokines and demonstrating anti-inflammatory properties in diabetic complications [21,22].

Our results align with and substantially extend previous reports on fullerene-based therapies in musculoskeletal disorders [5,10], while providing novel insights into their time-dependent effects on mechanokinetic parameters and oxidative stress dynamics. Our findings demonstrate that C_60_FAS treatment significantly improves two critical aspects of muscle function: maximal force generation (35–40% enhancement) and time to peak contraction (27–38% acceleration). These parameters are fundamental to the excitation-contraction-relaxation cycle. Precise coordination between motoneuron pool activation and muscle fiber recruitment is essential for optimal muscle function [23]. Injury-induced oxidative stress can disrupt this coordination by damaging voltage-gated sodium channels, thereby delaying action potential propagation [24]. Oxidative stress impairs mitochondrial ATP production and increases the generation of ROS [25]. Inflammatory processes following muscle injury are a source of ROS and contribute to the intensification of lipid peroxidation processes [26]. Therefore, in the present study, we evaluated changes in the blood of experimental animals in biochemical parameters.

The 25% lactate reduction may reflect either improved metabolic efficiency or attenuated inflammation, consistent with our observed decreases in SOD/CAT activity. It can also be suggested that a reduction in lactate levels may improve glycolytic ATP production, which is crucial for the detachment of myosin heads during rapid muscle contractions. This observation aligns with studies indicating that fullerenes stabilize mitochondrial membrane potential and reduce intracellular ROS production [27,28]. Such effects are clinically relevant for joint positioning accuracy, especially in fine motor tasks such as hand grasping, where millisecond delays in force generation can compromise movement precision [29,30,31,32].

The antioxidant defense system plays a central role in maintaining muscle homeostasis, particularly during post-traumatic recovery. SOD stands as the first line of protection against oxidative damage by catalyzing the conversion of superoxide radicals to hydrogen peroxide. Restoration of SOD activity achieved through fullerene C_60_ intervention demonstrates the compound’s capacity to disrupt the cycle of oxidative stress and mitochondrial dysfunction. Similarly, CAT works in concert with SOD by decomposing hydrogen peroxide into water and molecular oxygen, thereby preventing the formation of highly reactive hydroxyl radicals [33]. The normalization of CAT activity by 30–35% following fullerene C_60_ treatment indicates successful modulation of the intracellular redox environment.

The observed modulation of CAT activity following muscle injury and subsequent C_60_FAS treatment underscores the compound’s ability to restore redox equilibrium in damaged tissues. In the acute phase of muscle trauma, the increase in ROS activity triggers a compensatory upregulation of CAT. However, prolonged oxidative stress can exhaust this adaptive response [34]. Our experimental data align with these biochemical principles, as the initial post-injury CAT elevation was followed by a gradual decline. The 30–35% reduction in CAT activity suggests C_60_FAS partially supplants endogenous antioxidants by decreasing the H_2_O_2_ level through its electron-accepting carbon cage structure [26]. This dual action likely prevents the destructive feedback loop wherein H_2_O_2_ accumulation further inhibits CAT, exacerbating oxidative damage to lipids and proteins.

Morphological analyses provided structural correlates to these biochemical improvements. Injured muscle sections typically exhibit hallmark features of oxidative pathology [35]. These changes reflect the direct impact of ROS on cellular integrity. Samples from C_60_FAS-treated animals demonstrated markedly preserved myofibrillar architecture, with fewer necrotic fibers and reduced collagen deposition. This suggests that by maintaining CAT activity and reducing overall oxidative stress, the treatment safeguarded not only contractile proteins but also the extracellular matrix remodeling processes essential for functional recovery [36]. These morphological improvements further explain the biomechanical enhancements observed, such as increased maximum force developed and the time to reach maximum contraction, as intact sarcomeres and reduced fibrosis directly translate to better force transmission. Collectively, these findings position C_60_FAS as a modulator of both molecular and structural repair pathways in muscle tissue.

## 4. Materials and Methods

### 4.1. Material Preparation and Characterization

**Figure 5:**
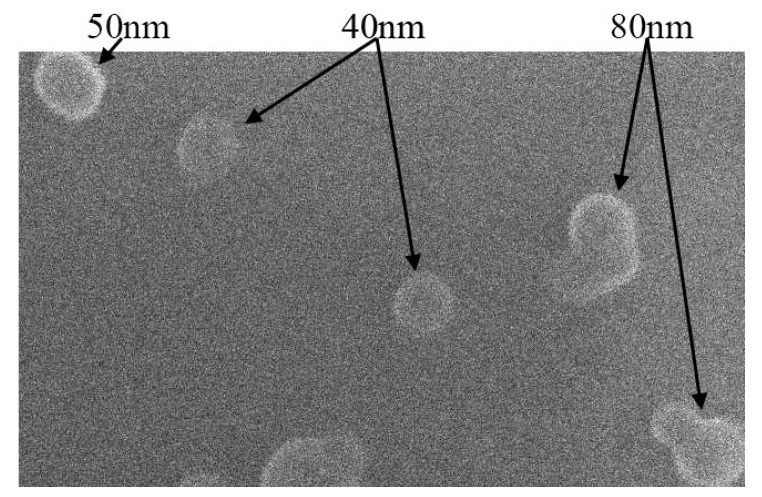
SEM image of C_60_ fullerene nanoparticles formed by thermal evaporation of the dispersant from the volume of a C_60_FAS (0.15 mg/mL) microdroplet at room temperature.

C_60_FAS was prepared with high purity exceeding 99.96% using an established ultrasonic-assisted phase transfer method from organic to aqueous media, as previously described [37]. The solution purity was rigorously verified through HPLC and GC/MS analyses to confirm the absence of residual impurities, including carbon black and organic solvents [38]. Comprehensive characterization of the C_60_FAS was performed using atomic force microscopy (AFM Solver Pro M system, NT-MDT, Optophase, Lyon, France) and dynamic light scattering (DLS, Zetasizer Nano-ZS90, Malvern Instruments, Malvern, UK). AFM imaging revealed two distinct molecular arrangements: individual C_60_ molecules appearing as discrete nanoscale features approximately 0.7 nm in height, and small nanoclusters measuring 1.3–2 nm in height, representing oligomeric aggregates. DLS measurements further confirmed the colloidal properties, showing a mean hydrodynamic diameter of 82 nm and a zeta potential of −23.9 mV, both indicating excellent solution stability. Finally, the scanning electron microscopy (SEM, FEI/Philips XL30 ESEM, Hillsboro, OR, USA) image of such nanoobjects (Figure 5) indicates their almost spherical shape. The maximum achievable concentration of stable C_60_ fullerene nanoparticles in aqueous medium using this preparation method was determined to be 0.15 mg/mL. This thorough characterization protocol ensures the reproducibility and quality of the C_60_FAS for subsequent biological applications.

### 4.2. Procedure and Experimental Animals

Male Wistar rats (100–120 g, 1-month-old) were used in the study. The animals were obtained from the vivarium of the ESC “Institute of Biology and Medicine” of Taras Shevchenko National University of Kyiv. The rats were housed in Plexiglas cages and kept in an air-filtered, temperature-controlled room (20–22 °C) under a 12-h light/12-h dark cycle. They received a standard pellet diet and water ad libitum. All animal procedures were approved by the Biomedical Ethics Committee of the ESC “Institute of Biology and Medicine” (Protocol #2, 2 September 2022) and conducted in accordance with the European Union Directive 2010/63/EU for the protection of animals used for scientific purposes. The study complied with ARRIVE guidelines.

The animals were randomly divided into 12 groups (*n* = 5 per group):

Groups 1–3: Control group (intact animals without muscle injury and/or C_60_FAS treatment), evaluated at 15, 30, and 45 days of the experiment, respectively.

Groups 4–6: Sham-treated group (animals administered C_60_FAS but not subjected to muscle injury), evaluated at 15, 30, and 45 days.

Groups 7–9: Muscle injury group (rats with injured gastrocnemius muscle), evaluated at 15, 30, and 45 days.

Groups 10–12: Muscle injury + C_60_FAS-treated group, evaluated at 15, 30, and 45 days.

**Figure 6:**
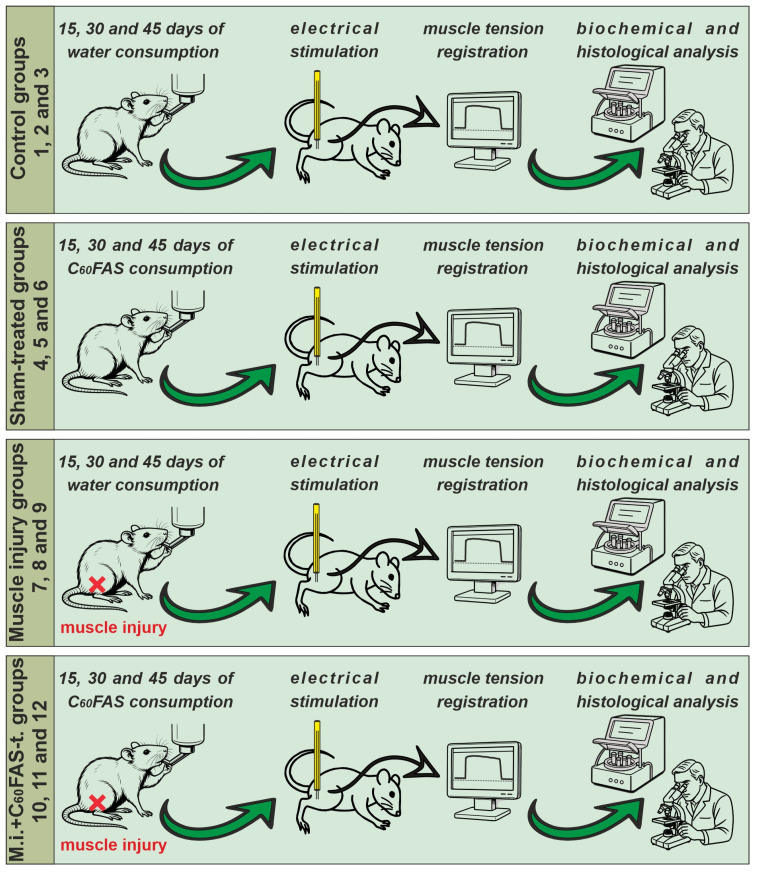
Schematic representation of the study. Groups 1–3: controls (water); 4–6: sham (C_60_FAS); 7–9: injury (water); 10–12: injury + C_60_FAS-treated (1 mg/kg/day). Evaluated at 15, 30, and 45 days. Protocol: oral administration → electrophysiology → biochemical/histological analyses. Parts of the figure were generated using DALL-E.

Animals in Groups 7–9 and 10–12 received oral C_60_FAS at a dose of 1 mg/kg daily after injury induction. This dose was selected based on its demonstrated efficacy in previous studies [5]. Muscle trauma was induced on day 0. We used independent animal cohorts for each time point (15, 30, and 45 days). Following the electrophysiological assessments, all animals underwent tensometric, biochemical, and histological analyses (see schematic representation of the study, Figure 6).

### 4.3. Operations and Electrical Stimulation Protocols

Animals were deeply anesthetized via intraperitoneal injection of Zoletil (40 mg/kg, Virbac, Carros, France). The skin over the right hindlimb was carefully dissected to expose the gastrocnemius muscle. The muscle was subjected to transverse incisions (1 mm depth) at three equidistant points. The skin wound was then sutured using synthetic absorbable copolymer suture material. Throughout the procedure and subsequent experiment, heart rate and body temperature were monitored.

At 15, 30, and 45 days post-injury, rats were reanesthetized (using the same protocol) for terminal experiments. The right gastrocnemius muscle was isolated from surrounding tissues, and its tendon was detached at the distal insertion point. The sciatic nerve was carefully separated and transected proximally, with all branches except those innervating the gastrocnemius muscle being cut. The animals were then secured to a platform, and the nerve was placed on a bipolar platinum wire electrode for electrical stimulation. The exposed muscle and nerve were immersed in paraffin oil within a skin-flap pool.

The gastrocnemius muscle was connected via the Achilles tendon to a servo-controlled muscle puller. Muscle tension was measured using semiconductor strain gauge resistors affixed to a stiff steel beam mounted on the linear motor’s moving part. The puller’s stiffness exceeded 0.06 N/mm, with length transient time constants ≤60 ms.

**Figure 7:**
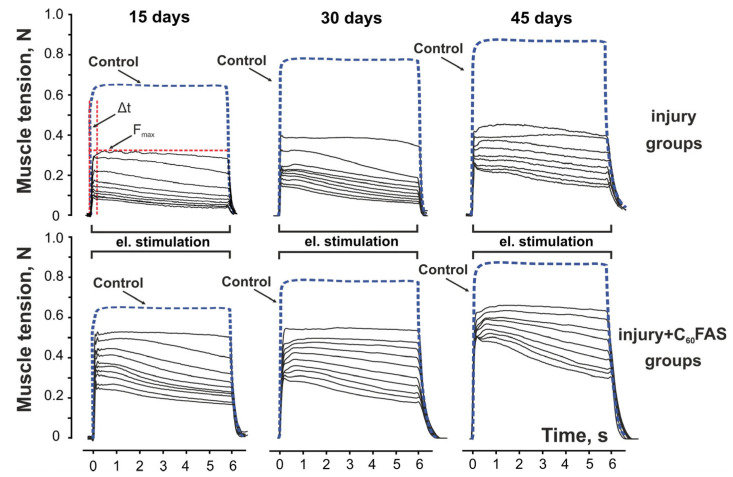
Representative recordings of 10 consecutive tetanic contractions in rat gastrocnemius muscle from different experimental groups. Data from one animal per group are shown as individual mechanograms, while control group data (blue line) represent averaged values across all control animals. Experimental groups include trauma (untreated injury) and trauma + C_60_FAS (injured animals treated with water-soluble C_60_ fullerene). Key parameters are indicated: Δt (time from stimulation onset to peak force), F_max_ (maximal force amplitude), and time points (15, 30, 45 days post-injury). The Δt and F_max_ parameters are indicated by vertical and horizontal red dashed lines, respectively. N—muscle tension (Newton).

Muscle contraction was induced using 10 intermittent high-frequency electrical stimulation series (6 s duration, separated by 4 s rest intervals) [5]. Each series consisted of 0.2-ms rectangular pulses at 50 s^−1^. Stimulus current was set to 1.3–1.4 times the motor threshold using a High-Power Bi-Phase Current Stimulator (Aurora Scientific Inc., Aurora, Canada). Examples of muscle tension patterns induced by above mentioned stimulations have been shown in Figure 7. Tensometric signals were digitized at 10 kHz and recorded using a Force Transducer System (Aurora Scientific Inc., Aurora, ON, Canada). Following the experiment, animals were euthanized via Nembutal overdose.

### 4.4. Biomechanical, Biochemical, and Histological Analyses

Muscle contraction parameters and blood biochemical markers were assessed at 15, 30, and 45 days post-injury.

The biomechanical parameters evaluated included:

*Maximal contraction force generation*: This global indicator of muscular dysfunction reflects impairments in both neural and myotonic components. Pathological reductions may result from disrupted synaptic currents in motoneurons, leading to impaired summation of transmembrane potentials and consequent alterations in action potential sequences that trigger contraction.

*Time to reach maximum force response*: This parameter reflects the muscle’s ability to generate rapid, ballistic movements. Changes in this metric indicate physiological dysfunction during maximal force generation tasks.

Biochemical analyses measured concentrations of lactate and oxidative stress markers (CAT, SOD) using clinical diagnostic analyzers (RNL-200 and JN-1101-TR2, Amsterdam, Netherlands).

For histological evaluation, gastrocnemius muscle samples were fixed in 10% formalin, paraffin-embedded, and sectioned at 5 µm. Sections were stained with hematoxylin and van Gieson picrofuchsin [39]. Digital micrographs were acquired at ×400 magnification using an Olympus BX41 microscope equipped with a C-5050 Zoom digital camera. Pathohistological profiles were assessed via light microscopy.

### 4.5. Statistical Analysis

Biomechanical data are presented as mean ± S.E.M. and were analyzed using two-way ANOVA with time and C_60_FAS treatment as factors. Between-group differences were assessed via Bonferroni post hoc tests (significance threshold: *p* < 0.05).

Biochemical data (mean ± S.E.M.) were also compared across time points and effects of C_60_FAS using two-way ANOVA followed by Bonferroni’s multiple comparison test (*p* < 0.05 considered significant). Data normality was verified using the Shapiro–Wilk test, and variance homogeneity was assessed via Levene’s test. All statistical analyses were performed using Origin 9.4 software (Origin Lab Corp., Northampton, MA, USA).

C_60_FAS treatment yielded large effect sizes in functional recovery (Cohen’s d = 1.0 for Fₘₐₓ) and moderate-to-large effects in oxidative stress reduction (d = 0.9 for CAT activity), suggesting clinically meaningful benefits. The large effect sizes observed in biomechanical parameters (e.g., Δt reduction) underscore C_60_FAS’s capacity to restore neuromuscular function. The 30–35% improvement in antioxidant markers (d > 0.8) further supports its role as a potent nanotherapeutic.

## 5. Conclusions

The present study provides evidence that C_60_FAS administration leads to notable improvements in neuromuscular function and redox balance in a rat model of muscle injury. Specifically, enhancements were observed across biomechanical, biochemical, and histological parameters, with improvements ranging from 35% to 40% compared to untreated injured animals. These findings suggest that C_60_FAS may have potential applications in mitigating muscle trauma effects, particularly in contexts requiring precise motor control, such as sports injuries or post-surgical rehabilitation. Given the preclinical nature of this research, further studies are necessary to fully elucidate the underlying mechanisms of action and to assess the efficacy and safety of C_60_FAS in clinical settings. Future investigations should focus on exploring the long-term effects, optimal dosing strategies, and potential interactions with other therapeutic agents. Additionally, expanding the scope to include diverse models of muscle injury could provide a more comprehensive understanding of C_60_FAS’s potential benefits. Overall, while these initial findings are promising, translating them into clinical practice will require rigorous validation through extensive research.

## Data Availability

The datasets used and/or analyzed during the current study are available from the corresponding author on reasonable request.

## Abbreviations

The following abbreviations are used in this manuscript:

AFM	Atomic force microscopy
ANOVA	Analysis of variance
CAT	Catalase
C_60_FAS	C_60_ fullerene aqueous solutions
DLS	Dynamic light scattering
GC/MS	Gas Chromatography/Mass Spectrometry
HPLC	High-Performance Liquid Chromatography
NLRP3	NOD-like receptor protein 3
NMDA	N-methyl-D-aspartate
NPs	Nanoparticles
ROS	Reactive oxygen species
S.E.M.	Standard error mean
SEM	Scanning electron microscopy
SOD	Superoxide dismutase
